# Safety, tolerability and pharmacokinetics of the oligomer modulator anle138b with exposure levels sufficient for therapeutic efficacy in a murine Parkinson model: A randomised, double-blind, placebo-controlled phase 1a trial

**DOI:** 10.1016/j.ebiom.2022.104021

**Published:** 2022-04-29

**Authors:** Johannes Levin, Nand Sing, Sue Melbourne, Amber Morgan, Carla Mariner, Maria Grazia Spillantini, Michal Wegrzynowicz, Jeffrey W. Dalley, Simon Langer, Sergey Ryazanov, Andrei Leonov, Christian Griesinger, Felix Schmidt, Daniel Weckbecker, Kai Prager, Torsten Matthias, Armin Giese

**Affiliations:** a MODAG GmbH, Wendelsheim, Germany; b Munich Cluster for Systems Neurology (SyNergy), Munich, Germany.; c Department of Neurology, Ludwig-Maximilians-University Munich, Germany; d Quotient Sciences, Mere Way, Ruddington Fields, Ruddington, Nottingham NG11 6JS, UK; e Department of Clinical Neurosciences, University of Cambridge, The Clifford Allbutt Building, Cambridge, CB2 0AH, UK.; f Laboratory of Molecular Basis of Neurodegeneration, Mossakowski Medical Research Institute, Polish Academy of Sciences, Warsaw, Poland; g Department of Psychology, University of Cambridge, Downing Street, Cambridge CB2 3EB, UK; h Department of Psychiatry, Hershel Smith Building for Brain and Mind Sciences, Addenbrooke's Hospital, Cambridge CB2 0SZ; i Department of NMR based structural Biology, Max Planck Institute for Biophysical Chemistry, 37077, Göttingen, Germany; j Cluster of Excellence “Multiscale Bioimaging: From Molecular Machines to Networks of Excitable Cells” (MBExC), University of Göttingen, Göttingen, Germany; k Center for Neuropathology and Prion Research, Ludwig-Maximilians-University Munich, Germany

**Keywords:** Multiple system atrophy, Parkinson disease, α-synuclein, Protein aggregation, Anle138b, Disease modification

## Abstract

**Background:**

Synucleinopathies such as Parkinson ´s disease (PD), Dementia with Lewy bodies (DLB) and Multiple System Atrophy (MSA) are characterized by deposition of misfolded and aggregated α-synuclein. Small aggregates (oligomers) of α-synuclein have been shown to be the most relevant neurotoxic species and are targeted by anle138b, an orally bioavailable small molecule compound which shows strong disease-modifying effects in animal models of synucleinopathies.

**Methods:**

Anle138b was studied in a single-centre, double-blind, randomised, placebo-controlled single ascending dose (SAD) and multiple ascending dose (MAD) study in healthy subjects. Eligible participants were randomly assigned (1:1 for sentinel subjects and 1:5 for main group) to placebo or anle138b (dose range 50 mg to 300 mg per day), respectively. In addition, the effect of food on the pharmakokinetics of anle138b in healthy subjects was examined in doses of 150 mg per day. Participants were randomized to treatment sequence (fed→fasted) or (fasted→fed). Treatment was administered orally in hard gelatine capsules containing either 10 mg or 30 mg of anle138b or excipient only. The primary endpoints were safety and tolerability, the secondary endpoint was pharmakokinetics. Data from all randomized individuals were evaluated. Clinicaltrials.gov-identifier: NCT04208152. EudraCT-number: 2019-004218-33.

**Findings:**

Between December 17^th^, 2019 and June 27^th^, 2020 196 healthy volunteers were screened and 68 participants were enrolled. Of these, all completed the study per protocol. There were no major protocol deviations. Adverse events in this healthy volunteer trial were mostly mild and all fully recovered or resolved prior to discharge. From baseline to completion of the trial no medically significant individual changes were observed in any system organ class. Already at multiple doses of 200 mg, exposure levels above the fully effective exposure in the MI2 mouse Parkinson model were observed.

**Interpretation:**

The favourable safety and PK profile of anle138b in doses resulting in exposures above the fully effective plasma level in a mouse Parkinson model warrant further clinical trials in patients with synucleinopathies.

**Funding:**

This study was funded by MODAG GmbH and by the Michael J. Fox foundation for Parkinson's Research.

**Research in Context**

**Evidence before this study**

We searched Pubmed up to July 31^st^, 2021, using the following terms: Synucleinopathy OR “Multiple system atrophy” OR “Parkinson Disease” AND “clinical trial” OR “treatment” AND “small molecule”. We identified no publication of a clinical stage development of a small molecule inhibiting α-synuclein as potential disease-modifying treatment for synucleinopathies. Besides the basic science literature about anle138b, there is one other publication describing the effect of another small molecule aiming at α-synuclein aggregates (UCB-0599). Both, UCB-0599 and anle138b have shown beneficial effects in animal models of synucleinopathies.

**Added value of this study**

This is the first clinical trial showing that the α-synuclein aggregation inhibitor anle138b has a favourable safety profile in healthy human volunteers. In addition, PK suggest that anle138b is well suited for long-term oral dosing in trials aiming at demonstrating efficacy.

**Implications of all evidence available**

Small molecules such as anle138b targeting pathological oligomers warrant further clinical trials in patients with synucleinopathies.

Alt-text: Unlabelled box

## Introduction

Synucleinopathies such as Parkinson´s disease (PD), Dementia with Lewy Bodies (DLB) or Multiple System Atrophy (MSA) are neurodenegerative disease characterised by aggregation of the protein α-synuclein in neurons and/or in oligodendrocytes (glial cytoplasmic inclusions), neuronal loss, and astrogliosis. The disease phenotype is dependent on the localization of pathological changes, which can predominantly affect the autonomic, nigro-striatal, ponto-cerebellar and cortical systems. MSA patients present dysautonomia combined with either predominant parkinsonism (MSA-P) or cerebellar ataxia (MSA-C).^1^ PD patients manifest predominantly with a hypokinetic-rigid phenotype,^2^ while patients with DLB show a mix of cognitive and motor disturbances.^3^ No effective therapies to slow disease progression are available.^4^ Inhibition of α-synuclein aggregation is a rational therapeutic intervention targeting a key pathophysiological process of synucleinopathies.^5^^,^^6^

The diphenyl-pyrazole anle138b (3-(1,3-benzodioxol-5-yl)-5-(3-bromophenyl)-1*H*-pyrazole) was discovered by a systematic drug development program.^7^ Anle138b represents a first-in-class compound that modulates toxic oligomers based on high-affinity binding to a structural epitope related to misfolding along the amyloidogenic pathway. It has been shown that compound binding destabilizes toxic oligomers, prevents the formation of oligomer pores in membranes and blocks prion-like propagation of α-synuclein aggregation.^7^, ^8^, ^9^, ^10^ Anle138b showed structure-dependent binding to pathological aggregates and strongly inhibited formation of pathological oligomers *in vitro* and *in vivo* for α-synuclein as well as for other disease-relevant amyloidogenic proteins such as prion protein, Abeta, and tau.^7^, ^8^, ^9^, ^10^, ^11^, ^12^, ^13^ The molecular mode of action of anle138b was also studied by all-atom molecular dynamics simulations on the multi-microsecond time scale.^14^ This work provides insight into the binding mechanism of anle138b to small oligomers and its ability to directly modulate these structures during the process of peptide aggregate formation. It was shown that anle138b reduces the overall number of intermolecular hydrogen bonds in oligomers, disfavors the sampling of the aggregated state, and remodels the conformational distributions within the small oligomeric peptide aggregates.^14^

In animal studies, anle138b showed a high oral bioavailability and excellent blood-brain barrier penetration leading to 5-fold higher levels of anle138b in the brain than in plasma.^7^ In a range of different mouse models, anle138b strongly inhibited oligomer accumulation, neuronal degeneration, and disease progression *in vivo*.^7^^,^^8^^,^^13^^,^^15^, ^16^, ^17^, ^18^

Here we investigated anle138b regarding safety, tolerability and pharmacokinetics (PK) in a first-in-human phase 1a clinical trial. Anle138b was administered in doses of up to 300 mg daily. It demonstrated excellent safety and tolerability profiles at all dose levels and reached significantly higher plasma levels in humans than those required for full therapeutic efficacy in MI2 mice, a recently established synucleinopathy rodent model.^18^

## Methods

### Study design

This was a single-centre, double-blind, randomised, placebo-controlled single ascending dose (SAD) and multiple ascending dose (MAD) study of anle138b in doses of up to 300 mg per day in healthy subjects. In addition, the effect of food (FES) on the PK of anle138b in healthy subjects was examined using single doses of 150 mg. Participants were recruited from Quotient Sciences (Nottingham, UK) volunteer database.

### Ethics

Approvals from the ethical review board (Wales REC2; IRAS PROJECT ID 273362; REC reference 19/WA/032719/WA/0327) and from the Medicines and Healthcare products Regulatory Agency (UK) (reference: CTA 51774/0001/001-0001) were obtained. This study was conducted in accordance with the protocol and with the following legislation: The updated version of the international Council for Harmonisation Good Clinical Practice (GCP) including the integrated Addendum E6, the Medicines for Human Use (Clinical Trials) Regulations including amendments No. 1928, 2984 and 941. In addition, the study was performed according to the ethical principles outlined in the World Medical Association Declaration of Helsinki and its amendments. Detailed descriptions of the study protocol have been published (www.clinicaltrials.gov, identifier NCT04208152; EU Clinical Trials Register, EudraCT Number 2019-004218-33; all last accessed July 28^th^, 2021). Written informed consent was obtained from all participants. The study data was monitored. A study monitor, independent of Quotient Sciences, was appointed to verify that the study was conducted in accordance with current GCP, regulatory requirements, the protocol and that the data were authentic, accurate and complete.

### Participants

Participants were healthy volunteers that needed to be 18 to 55 of age and able to understand the nature of the study and any risks involved in participation. They needed to be willing to cooperate and comply with the protocol restrictions and requirements and be capable and willing to give written informed consent. The study included healthy female volunteers with no childbearing potential as well as healthy male volunteers. Eligible participants needed to have a body mass index of (BMI) of 18·5 to 30·0 kg/m^2^ at screening and agreed to adhere to the contraception requirements defined in the study protocol. For participation in the food effect study, subjects were required to consume 90% of the US Food and Drug Administration (FDA)-approved high-fat breakfast in 25 minutes with dosing occurring at 30 min after the start of breakfast. Detailed inclusion and exclusion criteria are provided (supplement 1).

### Randomisation and masking

In SAD and MAD, eight participants were enroled per dosing cohort. Participants were randomly assigned to placebo (N=2) or to anle138b (N=6). Sentinel subjects (N=2) were randomized 1:1 and the main group (N=6) 1:5 for placebo or anle138b, respectively. A computer-generated randomisation schedule was used. All participants and study personnel directly interacting with participants were blinded to treatment assignment. The medication kits were numbered in a consecutive manner.

In FES, participants (N=12) were unblinded to treatment as this cohort did not include placebo, but were randomized in a 1:1 ratio to the sequences of treatment in a 2-way crossover design. Hence, 6 volunteers were randomized to the treatment sequence with prandial state first fed and then fasted, the remaining 6 volunteers to the fasted state first and then fed.

### Safe starting dose & exposure limit justification

This trial followed the recommendations of the EMA (European Medicines Agency) (EMEA/CHMP/SWP/28367/07 Rev. 1; 20 July 2017) and the FDA (Food and Drug Administration) (Guidance for Industry: Estimating the Maximum Safe Starting Dose in Initial Clinical Trials for Therapeutics in Adult Healthy Volunteers. U.S. Department of Health and Human Services Food and Drug Administration Center for Drug Evaluation and Research (CDER) July 2005) for dose finding in a first-in-human study. The safe starting dose was set based on toxicity studies in animals. In a 28-day toxicity study in rats, the no-observed-adverse-effect-level (NOAEL) was determined to be 50 mg/kg/day (human equivalent dose of 8·1 mg/kg using the FDA factor of 6·2 according to the FDA Guidance for Industry “Estimating the Maximum Safe Starting Dose in Initial Clinical Trials for Therapeutics in Adult Healthy Volunteers”). C_max_ǀexposure at 50 mg in males were 2460ǀ25600 and in females 2680ǀ21700 ng/mL ǀ ng*h/mL. In a 28-day cynomolgus monkey toxicity study, the NOAEL was 125 mg/kg/day (human equivalent dose of 40·3 mg/kg). C_max_ǀexposure at 125 mg/kg/day were 3170ǀ15700 (males) and 2720ǀ9720 (females) ng/mL ǀ ng*h/mL. Based upon these NOAELs a safe starting dose derived from the more sensitive species (rat) was calculated using a safety factor of 10 (i.e., 0·8 mg/kg), which corresponds to a dose of approximately 50 mg assuming a body weight of 60 kg. Based on these data, the dose range of the current trial was designed to start at 50 mg. Escalation between doses was planned to be flexible depending on emerging results but not to exceed an increment of 2-fold.

### Procedures

The trial medication was produced by Aptuit, an Evotec company, in Verona, Italy. Prior to production of the study medication (GMP batch) a formal formulation development was performed resulting in the production of a technical batch consisting of a semisolid in capsule formulation in size 00 capsule shells (2 dose strengths, 10 mg and 30 mg per capsule, respectively, and 1 placebo) with lauroyl macrogol-32-glycerides (Gelucire 44/14) as excipient. The capsule formulations were tested for: content uniformity, assay and impurities, appearance, water content, dissolution and XRPD (X-ray powder diffraction). The formal stability study was performed on the technical batch. Stability testing of two active batches and one placebo batch, packaged in typical primary packaging (HDPE bottle packs with desiccant) was conducted in parallel to the study.

Subjects were screened for enrollment in the study up to 28 days before dosing, they were admitted in the morning on the day before dosing (day -1) to the Quotient Sciences, clinical site, and remained on site until 48 h post last dose. A post study follow-up visit took place 5 to 7 days post last dose for safety & well-being monitoring. Screening of the volunteers included full physical examination, taking medical history and reviewing medical report, checking body weight and height to calculate body mass index (BMI) safety procedures such as safety bloods (haematology, clinical chemistry & virology, serum pregnancy test), 12 lead ECGs, vital signs (blood pressure, heart rate, and oral temperature), carbon monoxide breath tests, drug screen for drug of abuse, alcohol breath tests and urinalysis. Before subjects are admitted to the clinical unit, The Over Volunteering Prevention System (TOPS) were checked to ensure that each subject has not participated in a study at another site within at least 90 days of the dosing date.

As a safety precaution, sentinel dosing was performed for all SAD and MAD cohorts. In SAD, subjects received a single dose of anle138b or placebo after fasting from all food and drink (except water) for a minimum of 8 h prior to dosing. The dosing in the four SAD-cohorts were 50 mg, 100 mg, 200 mg and 300 mg of free anle138b equivalent, respectively. In the MAD part, participants received anle138b or placebo once daily (QD) for 7 days after fasting from all food and drink (except water) for a minimum of 8 h prior to dosing. The dosing in the three MAD-cohorts was 100 mg, 200 mg and 300 mg of free anle138b equivalent, respectively. Post dosing mouth and hand checks were conducted to ensure the capsules were swallowed. In-study decisions regarding continuation and dose escalation were made by the safety advisory committee (SAC) comprising the principal investigator, the sponsor's medical monitor and a PK expert. For dose escalation to proceed, data needed to be available from a minimum of 6 subjects per cohort with completed per protocol safety and PK assessments up to 48 h after dosing to ensure at least 4 subjects had received active IMP. The decision to proceed to the next higher dose level was based on safety, tolerability and available PK data to 48 h post-dose. The following data were required: Adverse events, vital signs, safety laboratory, ECG, physical examinations, plasma concentrations of anle138b with interim PK parameter estimations (T_max_, C_max_, AUC_(0-24)_, AUC_(0-tau)_, AUC_(0-last)_ and T_½_, where applicable). Data were provided to the SAC in accordance with Quotient Sciences’ standard operating procedure (SOP) on interim dose decision-making and dose escalation.

In the food effect study (FES), the effect of food on the PK of anle138b was explored using a single dose of 150 mg anle138b, a dose level that was previously deemed safe and well tolerated in the SAD and MAD cohorts. This dose was administered either i) after a standard FDA-approved high-fat breakfast or ii) in the fasted state i.e., fasted from all food and drink (except water) for a minimum of 8 h prior to dosing. In total, one cohort of 12 subjects was randomised in a 1:1 ratio to 2 treatment sequences (fed→fasted) or (fasted→fed). A minimum washout of at least 5 half-lives of anle138b between each dose was assured.

### Outcome measures

The primary objective was to assess the safety and tolerability of single (SAD) and multiple (MAD) ascending doses of anle138b to healthy subjects in the fasted state and to assess the safety and tolerability of single doses of anle138b in both the fasted and fed state (FES). To this end, adverse events (AEs), clinical laboratory tests, vital signs, electrocardiograms (ECGs), QT interval corrected for heart rate using Fridericia's formula (QTcF), and physical examination findings were recorded. Participants were instructed to report all potential AEs immediately to the on-site personnel and were assessed by a physician before each dose of study drug. In addition, a physical examination of the relevant body system was performed in the event that a subject reports any new symptoms or AEs. AEs were defined following standard criteria, which are described in the protocol. The detailed parameters and sampling schedule regarding haematology, clinical chemistry, ECG, blood pressure, heart rate and PK are provided in the supplement (supplement 3). Any clinically significant abnormality in these assessments, including changes from baseline, were needed to be reported as an AE.

Secondary outcome measures were the oral PK of single (SAD) and multiple (MAD) ascending doses of anle138b in the fasted state as well as the effect of co-administration with food on the PK of anle138b. To this end PK blood samples were taken at day 1 (and for MAD day 7) pre-dose and at 0·5, 1, 1·5, 2, 3, 4, 6, 8, 10, 12, 16 and 20 h post-dose, at days 2 to 6 pre-dose only (MAD) and 24, 30, 36 and 48 h post final dose. Analysis of anle138b in plasma was done at Aptuit, an Evotec company, in Verona, Italy. Time point of last quantifiable data was seen to increase with an increase in dose as follows: SAD: from the 50mg dose (subjects ranged between 8-24h) to the 300mg dose (subjects ranged between 36-48h). MAD: from the 100mg dose (subjects ranged between 8-36h) to the 300mg dose (subjects ranged between 20-48h). In FES, subjects ranged between 24-48h.

Data management was performed by Quotient Sciences using a validated electronic case report form (eCRF) database system and subjected to data consistency and validation checks. Data queries were raised within the study eCRF database by data management staff and resolved with the assistance of clinical staff.

AEs and medications were coded using the Medical Dictionary for Regulatory Activities (MedDRA) (v22·1). An independent coding review was performed within the Data Sciences department. Clinical chemistry and haematology data (and other safety laboratory data) were collected by a central laboratory (The Doctors Laboratory) and transferred electronically to Quotient Sciences. The list of parameters is provided in supplement 2. All demographic details and sample dates were cross-referenced with the corresponding data on the study database. Data was monitored by an external entity (Wirral Clinical Consultancy Ltd, Heswall, UK). Monitoring included the conduct of a site initiation visit, interim monitoring visits and a close-out visit. The database was closed after all queries had been resolved.

### Statistical analysis

The study was exploratory and no formal sample size calculation was made. Based on experience from previous studies of similar design, eight subjects per cohort were enrolled for parts 1 (SAD) and 2 (MAD) and a total of twelve subjects for part 3 (FES). Populations and analysis sets were determined for safety and PK data after database lock using the criteria defined in the reporting and analysis plan. The safety population and safety analysis set for SAD and MAD were defined after database lock but prior to study unblinding. Statistical analysis and production of summary tables, figures and listings for all safety data (AEs, vital signs, ECGs and safety laboratory assessments) including changes from baseline as required in this study were performed using the statistical package SAS (v9·4). Additional statistics was provided for PK-related data including coefficient of variation (CV%), geometric mean, geometric CV%, and the number n of subjects that were included in the natural logarithmic transformation). In SAD and MAD, dose proportionality was assessed. Formal statistical analysis was performed on the log-transformed PK parameters AUC_(0-last)_, AUC_(0-inf)_, and C_max_ for SAD (day 1), and AUC_(0-tau)_ and C_max_ for MAD (day 1 and day 7) to assess dose proportionality using the following power model: log_e_ (AUC or C_max_) = µ + βx log_e_ (dose). The estimate obtained for β is a measure of dose proportionality. The estimate of β together with its 90% confidence interval (CI) (βl, βu) was used to quantify the degree of non-proportionality. In MAD, formal statistical analysis was performed on the PK parameters AUC_(0-tau)_ and C_max_ to assess dose accumulation. Log-transformed AUC_(0-tau)_ and C_max_ was subjected to a mixed-effects model with treatment (dose level), day (day 1 or 7) and treatment-by-day interaction as fixed effects and subject as a random effect. The adjusted means obtained from the model, including differences for each comparison of interest and the associated 90% CIs, was back-transformed on the log scale to obtain adjusted geometric means, adjusted geometric mean ratios (GMRs) and 90% CIs of the ratio. The GMRs and 90% CIs were provided for each treatment and overall, i.e., day 7/day 1. In the FES, formal statistical analysis was performed on the PK parameters C_max_, AUC_(0-last)_ and AUC_(0-24)_ to assess the effects of food on anle138b. The PK parameters underwent a natural logarithmic transformation and were analysed using a mixed-effect model with terms for treatment (i.e., prandial state), period and sequence as fixed effects and subject nested within sequence as a random effect. Adjusted GMRs and 90% CIs for the adjusted GMRs for the comparison between fed and fasted states were provided where the ratios are defined as fed/fasted.

### Role of the funding source

MODAG GmbH served as sponsor of the trial, JL served as sponsor representative and as medical monitor. The sponsor funded data collection, analyses, and the initial interpretation of the data which were executed by Quotient Sciences, Nottingham, UK. The FES part was supported by a grant of the Michael J. Fox Foundation for Parkinson´s research. All authors had full access to the data. No medical writer or editor was employed. The decision to submit the manuscript was made by the authors with permission of MODAG GmbH.

## Results

**Figure 1:**
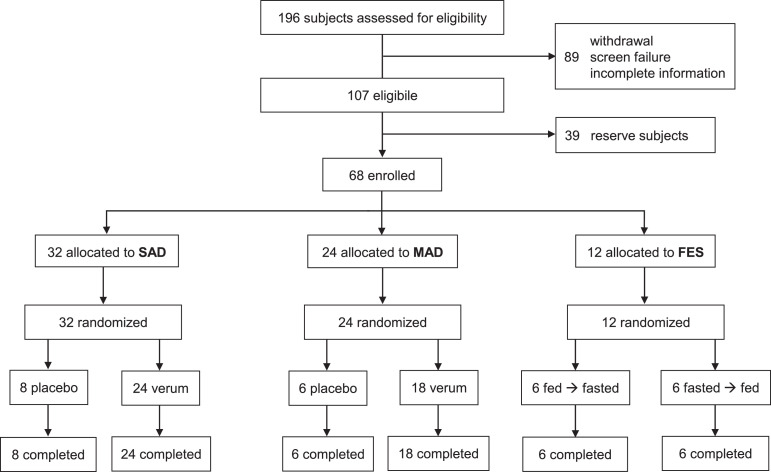
**Trial profile:** SAD: Single ascending dose. Dosing groups: 8 placebo participants & 6 participants per dose level verum (50, 100, 200, 300 mg). MAD: Multiple ascending dose. Dosing groups: 6 placebo participants & 6 participants per dose level verum (100, 200, 300 mg). FES: Food effect study. Groups: 6 participants each randomized to sequences. Sequence A: 150 mg fed → 150 mg fasted· Sequence B: 150 mg fasted → 150 mg fed.

Of 196 individuals assessed for eligibility, 89 failed screening, 39 served as reserve subjects and 68 were included in the study (Figure 1). Of the included participants, 32 subjects (4 dosing groups of 8) were included in the SAD part, 24 subjects (3 dosing groups of 8) in the MAD part and 12 subjects in the FES. Study-related admissions occurred between December 16^th^, 2019 and June 11^th^, 2020. In the SAD part, 8 participants received placebo. In the MAD part, 6 participants received placebo. All participants completed the study as planned per protocol. There were no dropouts or early discontinuations (Figure 1). Demographic characteristics of the study population at baseline were similar for all cohorts and groups (Table 1). Of 32 participants in the SAD part, 3 (9%) were female, all other participants including those in the MAD part and the FES were male. 100 % of the study medication was taken as scheduled in all groups.

**Table 1:** Demographic characteristics of study population. Data are mean (standard deviation) or - in case of gender - number (%). Abbreviations: BMI: Body mass index; *Single ascending doses of anle138b were studied in 4 dosing groups of 2 subjects receiving placebo and 6 subjects receiving anle138b, respectively. **Multiple ascending doses of anle138b were studied in 3 dosing groups of 2 subjects receiving placebo and 6 subjects receiving anle138b, respectively.

	Single Ascending Dose Cohorts*		Multiple Ascending Dose Cohorts**		Food Effect Cohort
	Placebo (N=8)	anle138b (N=24)	Placebo (N=6)	anle138b (N=18)	anle138b (N=12)
**Age [years]**	40 (12)	39 (13)	43 (9)	37 (11)	39 (9)
**Gender [% male]**	7 (88)	22 (92)	6 (100)	18 (100)	12 (100)
**Height [cm]**	169 (7)	177 (7)	179 (6)	176 (8)	179 (4)
**Weight [kg]**	72 (9)	80 (11)	82 (9)	76 (12)	84 (8)
**BMI [kg/m^2^]**	25·5 (2·5)	25·6 (2·3)	25·4 (2·0)	24·4 (2·8)	26·3 (2·7)

The primary objective of this trial were safety and tolerability. Treatment-emergent AEs were reported in comparable numbers in verum and placebo groups (Table 2). There was no dose dependency with regard to AE reporting. One event of vivid dreams in the placebo group of the MAD part and one event of headache in 200 mg anle138b of the MAD part were considered possibly related to the study medication in an assessment prior to unblinding. All other AEs were considered unrelated. All AEs but one were considered to be mild. The moderate AE was an episode of back pain and occurred in the MAD part at 100 mg anle138b. All AEs completely recovered. In only one of the dosing groups (subjects receiving placebo within the MAD part) the number of subjects with AEs exceeded the number of individuals without AEs. There were no clinically significant individual changes from baseline or notable trends in any safety assessment including laboratory values (clinical haematology, clinical chemistry or urinalysis), vital signs, physical examinations or ECG recordings in any subject included in the trial.

**Table 2:** Adverse events by system, outcome, grade, and attribution. Data are displayed as number of events (number of patients affected [%]). The exact nature of the adverse events within one system is provided in brackets.

	Single Ascending Dose Cohorts		Multiple Ascending Dose Cohorts		Food Effect Cohort
	Placebo (N=8)	anle138b (N=24)	Placebo (N=6)	anle138b (N=18)	anle138b (N=12)
**Adverse events: Number of patients with**					
**Adverse event(s)**	1 [12·5]	4 [16·7]	4 [66·7]	6 [33·3]	5 [41·7]
**No adverse event**	7 [87·5]	20 [83·3]	2 [33·3]	12 [66·7]	7 [58·3]
**Adverse events by system**					
**Nervous system disorders (headache, paraestesia)**	0	2 [8·3]	2 [33·3]	4 [16·7]	3 [25]
**Eye disorders (ocular hyperaemia, vision blurred)**	0	1 [4·2]	0	1 [5·6]	0
**Gastrointestinal disorders (diarrhea, nausea)**	1 [12·5]	0	1 [16·7]	1 [5·6]	0
**Infections and infestations (Nasopharyngitis, rhinitis)**	0	1 [4·2]	0	1 [5·6]	0
**Musculosceletal and connective tissue disorders (Back pain)**	0	1 [4·2]	0	2 [11·1]	0
**General disorders and administration site conditions (Chest discomfort, fatigue, medical device site reaction)**	0	0	1 [16·7]	1 [5·6]	1 [8·3]
**Psychiatric disorders (abnormal dreams)**	0	0	1 [16·7]	0	0
**Injury, poisoning and procedural complications (procedural dizziness)**	0	0	0	0	2 [16·7]
**Adverse events by outcome (number of events)**					
**Recovered (%)**	1 (100)	5 (100)	5 (100)	10 (100)	6 (100)
**Missing / Unknown**	-	-	-	-	-
**Adverse events by grade (number of events)**					
**Mild (%)**	1 (100)	5 (100)	5 (100)	9 (90)	6 (100)
**Moderate (%)**	0	0	0	1 (10)	0
**Adverse events by attribution to study treatment (number of events)**					
**Definite**	0	0	0	0	0
**Probable**	0	0	0	0	0
**Possible (%)**	0	0	1 (20)	1 (10)	0
**Not related(%)**	1 (100)	5 (100)	4 (80)	9 (90)	6 (100)

**Figure 2:**
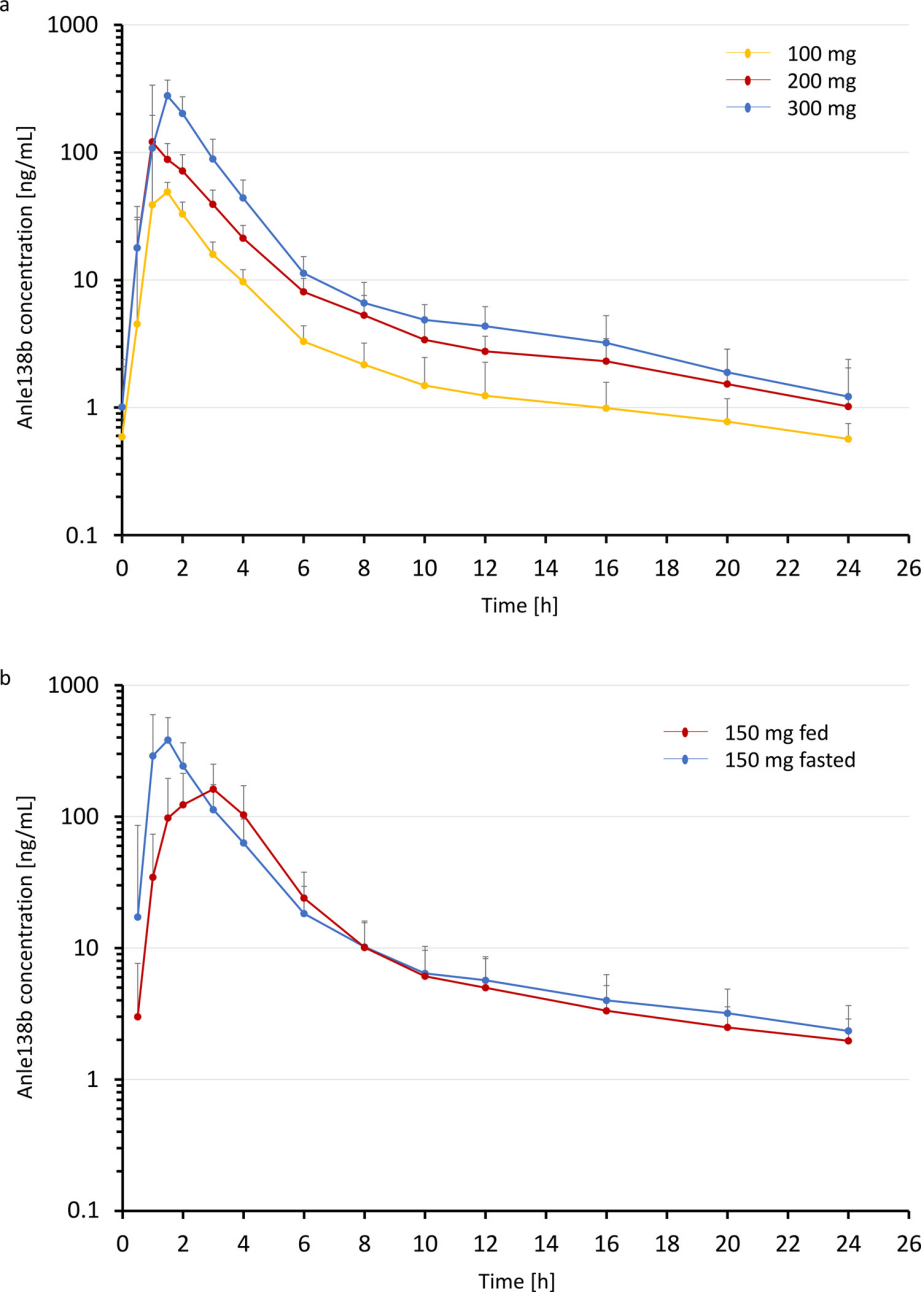
**Pharmakokinetic profile of anle138b:** Geometric Mean (CV%) of anle138b in healthy volunteers. a) Data from day 7 of multiple oral administration of anle138b in capsule form from N=6 participants per dosis group are shown. b) Data following single oral administration of anle138b in capsule form from N=12 participants in the fed or in the fasted state, respectively, are shown.

The seconday objective was PK. Following oral administration of anle138b in capsule form, plasma concentrations of anle138b became quantifiable at 0·5-1 hours post-dose in all subjects and remained quantifiable for 24 to 48 hours post-dose. This was seen in SAD, in FES at the dosing days and in MAD both at day 1 and at day 7 where detailed PK data were gathered (Table 3). Maximum concentrations were achieved between 0·5 and 2 hours post-dose (median T_max_ of 1 to 1·5 hours post-dose). T_max_ was unaffected by multiple dosing (Figure 2a). In SAD, C_max_ values increased in a greater than dose proportional manner, by a factor approximately 3·0 over the 50 to 200 mg dose range and generally consistant with proportionality from 200 to 300 mg (Supplement Figure 1a). In MAD, on day 1, C_max_ values increased in a greater than dose proportional manner over the 100 to 300 mg dose range, again by a factor of approximately 3·0 for a 2-fold increase in dose (Supplement Figure 1b). Repeated administration of anle138b capsules in the fasted state resulted in reductions in C_max_ and AUC exposures: Compared to day 1, for AUC_(0-tau)_ in the 100 mg group at day 7 an accumulation factor of 0·54 was found, hence the exposure of anle138b did not increase but decrease from day 1 to day 7. Therefore, the accumulation factor is <1. In the 200 mg group the accumulation factor was 0·34 and in the 300 mg group the accumulation factor was 0·29. Across all groups repeated daily dosing of anle138b resulted in an accumulation factor of AUC_(0-tau)_ of 0·39 while the accumulation factor of C_max_ was 0·38. These decreases with multiple dosing are in line with the results seen in repeat-dosing animal studies. The decreased exposure is likely a result of the induction of metabolizing CYP enzymes, particularly CYP1A2. Potential for drug-drug interactions resulting from CYP-induction has not been studied in this trial. A reduction of levels of anle138b was also observed for trough (i.e., pre-dose) levels. Of note, steady state exposure appeared to be reached by day 5 (supplement Figure 2). The elimination half-life of anle138b after single dosing was variable. T_½_ was 3·9 hours in the 50 mg cohort and 10·8 h and 12·8 h, respectively, in the 100 mg and 200 mg cohorts. T_½_ increased to 16·2 h in the 300 mg group. The variability in T_½_ over the dosing range is suspected to be due to differences in the time of last quantifiable concentrations between subjects, leading to concentrations falling below the limit of quantification during the later portions of the profiles, leading to an inaccurate characterisation of the true terminal elimination phase especially in the 50 mg cohort. Therefore, the true T_½_ of anle138b in human plasma is at least 10 h. Multiple dosing also tended to result in a decrease in half-life compared to the single ascending doses in Part 1 of the study, except for T_½_ in the 200 mg group that was about 10 hours and, thus, largely similar in MAD and in SAD. Overall, the PK data showed good bioavailability of anle138b and a practical elimination half-time of ∼12 h. Potential therapeutic exposure (based upon nonclinical *in vivo* models, see supplement figure 4) was already achieved after single 100 mg dose of anle138b. With increased doses of 200 or 300 mg, exposure levels were increased correspondingly, without any safety concerns. In the FES part, maximum concentrations were achieved between 1- and 2-hours post-dose (median T_max_ of 1·25 hours post-dose) in the fasted state and between 1·5 and 3 hours post-dose (median T_max_ of 3·00 hours post-dose) in the fed state (Figure 2b). A change in prandial state from fasted to fed resulted in approximately 74% (90% CI: 61%, 88%) of the dose being bioavailable in the fed state compare to the fasted state. C_max_ was decreased by about half with food, in line with the delay in T_max_. Inter-subject variability with relation to exposure (C_max_ and AUC) was moderate across both regimens. The elimination half-life of anle138b was only slightly higher in the prandial state, with geometric mean values of 11·3 and 15·2 hours, following administration in the fasted and fed regimens respectively. Overall, the PK data showed good bioavailability of anle138b following both fasted and fed conditions and the food effect on AUC was considered clinically insignificant in light of the variation in individual patient values.

**Table 3:** Pharmakokinetic profile of anle138b: Geometric Mean (CV%) key pharmacokinetic parameters of anle138b in healthy volunteers following single oral administration of anle138b in capsule form. For T_max_ Median (range) is shown. Abbreviations: T_max_: Time to maximum peak; C_max_: Maximum concentration; AUC: Area under the curve (= exposure); T_1/2_: Plasma half-life; h: Hour; ng: Nanogram; ml: Millilitre; NA: Not applicable.

Treatment	Sampling day	T_max_* (h)	C_max_ (ng/mL)	AUC_(0-24)_ (ng·h/mL)	T_1/2_ (h)
**Single Ascending Dose Study**					
**Cohort A** **50 mg**	Dosing day	1·00 (0·50–2·00)	54·3 (58·5)	113 (66·0)	3·9 (201·3)
**Cohort B** **100 mg**	Dosing day	1·50 (0·50–2·00)	156 (30·5)	366 (23·4)	10·8 (57·6)
**Cohort C** **200 mg**	Dosing day	1·50 (1·00–2·00)	458 (56·3)	1090 (55·8)	10·3, 15·8 [N=2]
**Cohort D** **300 mg**	Dosing day	1·50 (1·50–2·00)	704 (49·5)	1650 (48·6)	16·2 (32·3)
**Multiple Ascending Dose Study**					
**Cohort A** **100 mg**	Dosing day 1	1·00 (1·00–1·50)	135 (38·9)	261 (42·5)	NA
	Dosing day 7	1·25 (1·00–1·50)	70·9 (55·4)	141 (40·7)	4·2 (119·6) [N=4]
**Cohort B** **200 mg**	Dosing day 1	1·50 (1·00–2·02)	447 (58·0)	905 (60·7)	NA
	Dosing day 7	1·00 (1·00–1·50)	128 (55·5)	308 (34·1)	9·5 (78·8)[N=5]
**Cohort C** **300 mg**	Dosing day 1	1·50 (1·50–2·00)	910 (94·9)	2210 (81·1)	NA
	Dosing day 7	1·50 (1·00–1·50)	307 (55·9)	633 (37·3)	6·06, 6·07 [N=2]
**Food Effect Study**					
**150 mg fasted**	Dosing day	1·25 (1·00–2·00)	442 (58·7)	896 (54·7)	11·3 (17·4)[N=3]
**150 mg fed**	Dosing day	3·00 (1·50–3·08)	196 (48·6)	641 (49·0)	15·2 (28·3)[N=7]

## Discussion

All approved treatment options for synucleinopathies are purely symptomatic. This constitutes an urgent medical need to develop disease-modifying treatments for these conditions that include DLB, MSA and PD.^19^ α-Synuclein aggregation has become a major target for therapy development. The rationale for this strategy is based on the emerging central role of prion-like amplification and cell-to-cell propagation pathological α-synuclein aggregates as well as aggregate-mediated toxicity of α-synuclein in the pathogenesis of synucleinopathies.^5^^,^^6^

New therapeutics aiming at disease-modification in synucleinopathies in the clinical development stage can currently be grouped regarding mode of action as I) interventions preventing α-synuclein aggregation or dissolving aggregates, II) interventions that reduce α-synuclein expression^20^ or affect α-synuclein metabolism and clearance (LRRK2 inactivation and reduction of LRRK2 gene expression,^21^ c-Abl- inhibition, mTOR–inhibition, and increase of lysosomal activity via stimulation of GBA.^5^^,^^6^^,^^22^), and III) interventions that target other processes involved in neurodegeneration such as neuroinflammation, neuronal cell death or iron metabolism.^5^^,^^6^^,^^19^^,^^23^ As formation of pathological α-synuclein aggregates is a non-physiological process, these aggregates are not found in the healthy brain. Therefore, strategy I) has advantages over strategies II) and III) as these involve modification of physiological cellular and systemic targets that might lead to inherent side effects. Approaches in clinical development following strategy I) include the development of monoclonal antibodies and small molecules. In contrast to antibodies, small molecules have the potential to efficiently pass the blood-brain barrier and directly target toxic intracellular α-synuclein oligomers where they are formed. Moreover, antibodies once bound to the target have the potential to trigger inflammation.^24^

To date two small molecules targeting α-synuclein aggregation have reached the clinical state of development: Anle138b and UCB-0599 (previously known as NPT200-11).^25^ These small molecule compounds have shown to directly target aggregation of α-synuclein and have been tested in a range of animal models of synucleinopathies.^17^^,^^18^^,^^25^ Notably, anle138b has shown to be effective also in the symptomatic disease state and restore altered dopamine release in the striatum.^16^^,^^18^ In animals, anle138b has been shown to efficiently cross the blood-brain barrier.^7^ The missing piece of information for further development of anle138b in efficacy trials in human patients is safety and tolerability of anle138b in humans at exposure levels that are matching full therapeutic efficacy in animal experiments. The dose-response information of anle138b to restore striatal dopamine release in a highly innovative animal model of PD is shown in the supplement (supplement 4).^18^

In our first-in-human phase 1a study, we generated data of safety, tolerability and PK of anle138b in human healthy volunteers. Demographic and clinical baseline characteristics of the recruited volunteers are consistent with similar healthy volunteer studies. With multiple dosing at a daily dose of 200 mg we reached exposure levels of ≥300 ng*h/ml (AUC_0-24_), the plasma level required for full efficacy in a relevant PD mouse model (supplement 4). With the highest dose tested (300 mg) more than twice this exposure was achieved. At all tested doses (up to 300 mg) we observed excellent tolerability and no compound-related side effects in this study (Table 2).

Limitations: This is a standard phase 1a study in healthy volunteers not allowing for the evaluation of potential off-target effects, target-specific side effects or disease-specific effects on PK in a patient population. Moreover, no efficacy assessment was possible in this healthy population. Finally, the cohort studied included significantly more men than women. This is due to the exclusion of women of childbearing potential (WOCBP) which is in line with recommendations for phase 1 trials of novel compounds. For future trials in patients with neurodegenerative diseases exclusion of WOCBP will have less impact due to the different age distribution. Moreover, this exclusion criterion will be reassessed based on available data and risk/benefit cosiderations.

Conclusion: The safety profile of anle138b established in these small cohorts is encouraging. Further, longer and large-scale trials will help to establish the benefit risk profile of anle138b in patients with synucleinopathies.

## Contributors

Johannes Levin: Design and management of the study, sponsor-delegated medical monitor, verification of underlying data, writing of manuscript including tables and figures.

Nand Sing: Design and conduction of the study, principal investigator, verification of underlying clinical data, revision of manuscript, tables and figures.

Sue Melbourne: Design and conduction of the study, revision of manuscript, tables and figures.

Amber Morgan: Design and conduction of the study, lead pharmacokineticist, verification of underlying pharmacokinetics data, revision of manuscript, tables and figures.

Carla Mariner: Design and conduction of the study, verification and interpretation of pharmacokinetics data, revision of manuscript, tables and figures.

Maria Grazia Spillantini: Animal experiments, revision of manuscript, tables and figures.

Michal Wegrzynowicz: Animal experiments, revision of manuscript, tables and figures.

Jeffrey Dalley: Animal experiments, revision of manuscript, tables and figures.

Simon Langer: Animal experiments, revision of manuscript, tables and figures.

Sergey Ryazanov: Planning and drug supply for animal experiments. Revision of manuscript, tables and figures.

Andrei Leonov: Planning and drug supply for animal experiments. Revision of manuscript, tables and figures.

Christian Griesinger: Planning and drug supply for animal experiments. Revision of manuscript, tables and figures.

Felix Schmidt: Study management, revision of manuscript, tables and figures.

Daniel Weckbecker: Study management, revision of manuscript, tables and figures.

Kai Prager: Design of the study, revision of manuscript, tables and figures.

Torsten Matthias: Design of the study, revision of manuscript, tables and figures.

Armin Giese: Design and management of the study, verification of underlying data, writing of manuscript including tables and figures.

All authors read and approved the final version of the manuscript.

## Data sharing statement

Data collected for the study, including deidentified participant data and a data dictionary defining each field in the set, will be made available to others upon formal request and with a signed material transfer agreement. Upon formal request and with a signed material transfer agreement related documents will be available (e.g., study protocol, statistical analysis plan, informed consent form). The data may be requested upon publication. The data will only be shared via individual secured network connections.

## Declaration of interests

Johannes Levin reports part-time employment by MODAG GmbH and a grant of the Michael J Fox Foundation for Parkinson´s Research. In addition, he reports speaker fees from Bayer Vital, Biogen and Roche, consulting fees from Axon Neuroscience and Biogen, author fees from Thieme medical publishers and W. Kohlhammer GmbH medical publishers, all outside the submitted work. He is beneficiary of the phantom share program of MODAG GmbH. In addition, he is inventor in a patent “Pharmaceutical Composition and Methods of Use” (EP 22 159 408.8) filed by MODAG GmbH.

Nand Sing reports employment by Quotient Sciences which was contracted under a services agreement to perform activities related to the design, conduct, and reporting of this clinical study.

Sue Melbourne reports employment by Quotient Sciences which was contracted under a services agreement to perform activities related to the design, conduct, and reporting of this clinical study.

Amber Morgan reports employment by Quotient Sciences which was contracted under a services agreement to perform activities related to the design, conduct, and reporting of this clinical study.

Carla Mariner reports employment by Quotient Sciences which was contracted under a services agreement to perform activities related to the design, conduct, and reporting of this clinical study.

Maria Grazia Spillantini reports funding for this study from Parkinson´s UK consulting fees from Mission therapeutics and speaker fees from CASMA and BIAL, outside this work. She is in the Scientific Advisory Board of the Tau consortium sponsored by the Rainwater foundation.

Michal Wegrzynowicz reports no conflicts of interest.

Jeffrey Dalley reports grants held with Glaxo Smith Kline and Boehringer Ingelheim Pharma GmbH & Co. KG.

Simon Langer reports no conflicts of interest.

Sergey Ryazanov reports part-time employment by MODAG GmbH. In addition, he is inventor in a patent “water-soluble derivatives of 3,5-diphelyl-diazole compounds” (PCT EP 16/081084 etc.) licensed to MODAG GmbH, and a patent “new drugs for inhibiting aggregation of proteins involved in disease linked to protein aggregation and/or neurodegenerative diseases” (EP 2307381 etc.), which includes the compound anle138b, licensed to MODAG GmbH and a patent PCT/EP2020/082778 “Novel compounds for the diagnosis, treatment and prevention of disease associated with the aggregation of alpha-synuclein”. In addition, he is beneficiary of the phantom share program of MODAG GmbH.

Andrei Leonov reports part-time employment by MODAG GmbH. In addition, he is inventor in a patent “water-soluble derivatives of 3,5-diphelyl-diazole compounds” (PCT EP 16/081084 etc.) licensed to MODAG GmbH, and a patent “new drugs for inhibiting aggregation of proteins involved in disease linked to protein aggregation and/or neurodegenerative diseases” (EP 2307381 etc.), which includes the compound anle138b, licensed to MODAG GmbH and a patent PCT/EP2020/082778 “Novel compounds for the diagnosis, treatment and prevention of disease associated with the aggregation of alpha-synuclein”. In addition, he is beneficiary of the phantom share program of MODAG GmbH.

Christian Griesinger reports consultancy for and being a shareholder of MODAG GmbH. In addition, he is inventor in a patent “water-soluble derivatives of 3,5-diphelyl-diazole compounds” (PCT EP 16/081084 etc.) licensed to MODAG GmbH, and a patent “new drugs for inhibiting aggregation of proteins involved in disease linked to protein aggregation and/or neurodegenerative diseases” (EP 2307381 etc.), which includes the compound anle138b, licensed to MODAG GmbH and a patent PCT/EP2020/082778 “Novel compounds for the diagnosis, treatment and prevention of disease associated with the aggregation of alpha-synuclein”.

Felix Schmidt reports employment by MODAG GmbH. In addition, he is inventor in a patent “water-soluble derivatives of 3,5-diphelyl-diazole compounds” (PCT EP 16/081084 etc.) and is inventor in a patent “Novel Compounds for the Diagnosis, Treatment and Prevention of Diseases Associated with the Aggregation of alpha-Synuclein” (PCT/EP2020/082778) both licensed to MODAG GmbH. In addition, he is beneficiary of the phantom share program of MODAG GmbH.

Daniel Weckbecker reports employment by MODAG GmbH. In addition, he is inventor in a patent “Novel Compounds for the Diagnosis, Treatment and Prevention of Diseases Associated with the Aggregation of alpha-Synuclein” (PCT/EP2020/082778) licensed to MODAG GmbH. In addition, he is beneficiary of the phantom share program of MODAG GmbH.

Kai Prager reports part-time employment by MODAG GmbH. In addition, he is beneficiary of the phantom share program of MODAG GmbH.

Torsten Matthias reports being CEO of MODAG GmbH and being a shareholder of MODAG GmbH. In addition, he is beneficiary of the phantom share program of MODAG GmbH.

Armin Giese reports employment by and being a shareholder of MODAG GmbH. In addition, he is inventor in a patent “water-soluble derivatives of 3,5-diphelyl-diazole compounds” (PCT EP 16/081084 etc.) licensed to MODAG GmbH, and a patent “new drugs for inhibiting aggregation of proteins involved in disease linked to protein aggregation and/or neurodegenerative diseases” (EP 2307381 etc.), which includes the compound anle138b, licensed to MODAG GmbH and a patent PCT/EP2020/082778 “Novel compounds for the diagnosis, treatment and prevention of disease associated with the aggregation of alpha-synuclein” and he is inventor in a patent “Pharmaceutical Composition and Methods of Use” (EP 22 159 408.8) filed by MODAG GmbH. In addition, he is beneficiary of the phantom share program of MODAG GmbH.

## Appendix Supplementary materials

Image, application 1

Image, application 2
