# Supplementary material for: Safety, tolerability and pharmacokinetics of the oligomer modulator anle138b with exposure levels sufficient for therapeutic efficacy in a murine Parkinson model: A randomised, double-blind, placebo-controlled phase 1a trial
Source: eBioMedicine. 2022 Apr 29;80:104021. doi: 10.1016/j.ebiom.2022.104021 (PMC9065877; doi:10.1016/j.ebiom.2022.104021)
Supplement: Supplementary file 1 [file mmc1.docx]

Supplement 4: Animal experiments

Methods:

An anle138b pharmacodynamics dose-response study was performed using MI2 mice, a novel model of α-synucleinopathy characterized by a progressive PD-like phenotype including accumulation of α-synuclein inclusions in nigrostriatal dopaminergic neurons and impairment of striatal dopamine (DA) release (Wegrzynowicz et al., 2019). The research was carried out in accordance with the Animals (Scientific Procedures) Act 1986 Amendment Regulations 2012 after the review by the University of Cambridge Animal Welfare and Ethical Review Body (AWERB), under the project license 70/8383. Anle138b treatment was commenced at 6 months, an age when impairment of striatal DA release is already observed in MI2 mice, but prior to nigral neuronal loss. MI2 animals (total of 55) were divided into 7 gender-balanced treatment groups, containing between 7 and 9 animals, that were fed with standard mouse chow (ssniff Spezialdiäten GmbH) supplemented with different doses of anle138b (0 (n=9), 0·008 (n=7), 0·025 (n=7), 0·074 (n=7), 0·222 (n=8), 0·667 (n=8), and 2 (n=9) grams of compound per kg of food, respectively), provided at libitum. Assuming a daily food consumption of 0·15 g food pellets per g body weight, the doses were considered to correspond to 1·2, 3·75, 11·1, 33·3, 100, and 300 mg/kg/day. After 2 months of treatment (at the age of 8 months), striatal release of DA was analysed using *in vivo* microdialysis, as previously reported (Wegrzynowicz et al., 2019). Briefly, 24 hours prior to the experiment, a mouse received an injection of an analgesic, Carprieve, was anesthetized with isoflurane and a microdialysis guide cannula CMA7 (CMA Microdialysis) was stereotactically implanted into the brain, so that its tip reached the striatal surface (+0·8 anteroposterior and +2·1 lateral relative to bregma, and -2·3 below the skull surface according to (Paxinos and Franklin, 2004)). On the next day, after full recovery of the animal, microdialysis probe CMA7 (membrane: 0·24 mm × 2 mm, 6 kDa cut-off; CMA Microdialysis) was inserted into the cannula, and freely-moving mouse was infused with artificial Cerebro-Spinal Fluid (aCSF), composed of 140 mM NaCl, 7·2 mM glucose, 3 mM KCl, 1 mM MgCl_2_, 1·2 mM CaCl_2_, 1·2 mM Na_2_HPO_4_, 0·27 mM Na_2_HPO_4_, pH 7·4 at a flow rate of 2 µl/min. Following 30 min equilibration period, the microdialysates were collected every 20 min into the tubes prefilled with 5 µl of 0·2 M perchloric acid. After 60 min, physiological aCSF was switched to high-potassium aCSF containing 93 mM NaCl and 50 mM KCl, in order to induce release of DA from nigrostriatal synapses. 60 min later, high potassium aCSF was switched back to physiological aCSF. Individual samples were frozen in dry ice immediately after collection and stored at -80°C before DA measurements. At the end of the experiment, the mouse was sacrificed and the position of the probe insertion was inspected in the isolated brain. DA levels in the microdialysates were measured by high performance liquid chromatography with electrochemical detection, as described in (Garcia-Reitböck et al., 2010). Briefly, samples were resolved with a Hypersil BDS C18 reversed phase column (100 × 4·6 mm, 3 µm particle size, 130 Å pore size; Phenomenex) at a flow rate of 1 ml/min, in a mobile phase composed of citric acid (31·9 g/l), sodium acetate (2 g/l), octanesulfonic acid (460 mg/l), EDTA (30 mg/l) and methanol (15%), pH 3·6. DA detection was performed by redox oxidation using ESA 5014 analytical cell and ESA Coulochem II detector (Thermo Fisher Scientific), with electrodes set at -200 mV (reducing) and +250 mV (oxidizing). The obtained chromatograms were analysed with Chromeleon Chromatography Data System (V 6·2; Dionex).

The number of animals allocated to the treatment groups was based on the previous dopamine release microdialysis experiments performed in this model following anle138b treatment, but was also adapted to the number of animals in individual litters, since the animals from the same litter and of the same sex were kept together in individual cages and treated with the same dose of the drug. Only mice that didn’t display any visible signs of disease or injury were included in the experiments. Over the whole course of the experiment, the animals were kept in the animal facility at the University of Cambridge under the strict supervision of experienced veterinary technicians. All the health issues were immediately reported to the experimenter, and such animals were excluded from the experiment and sacrificed. In total, at different stages of the experiment, 5 animals were lost due to various reasons: spontaneous death before anle138b treatment commencement (1 animal in 11·1 mg/kg/day group), health issues (1 animal in 100·4 mg/kg/day group and 1 animal in 300 mg/kg/day group), or inability to recover from anesthesia following surgical implantation of microdialysis cannula (one animal in 11·1 mg/kg/day group and 1 animal in 33·3 mg/kg/day group). All the remaining mice (n=50) successfully underwent microdialysis procedure and all the fractions collected from these mice were measured by HPLC, and all the data obtained from HPLC assay were included in the final analysis. For each experimental group, the exact number of animals that were included in the final analysis was as follows: 0 mg/kg/day, n=9; 1·2 mg/kg/day, n=7; 3·75 mg/kg/day, n=7; 11·1 mg/kg/day, n=5, 33·3 mg/kg/day, n=7; 100 mg/kg/day, n=7; and 300 mg/kg/day, n=8.

Due to low number of units (usually 2 cages per experimental group) no formal randomization was used to allocate animals to the experimental groups. Allocation was done arbitrary by the experimenter, but at the time of allocation, the experimenter didn’t have any detailed knowledge about individual animals, apart from most basic information (date of birth, sex and parents). In order to minimize potential confounders, the following steps were taken: (i) due to different dates of birth of the mice used in the experiment (between April 21^st^ 2017 and June 26^th^ 2017), the treatment of individual cages commenced at different dates, spanning over two months, and was performed in random rather than sorted order (it started with placebo cage, then 300 mg/kg/day, 33·3 mg/kg/day, 11·1 mg/kg/day, 100 mg/kg/day, 1·2 mg/kg/day, placebo, 3·75 mg/kg/day, 33·3 mg/kg/day, 300 mg/kg/day, 100 mg/kg/day, 11·1 mg/kg/day, 300 mg/kg/day, 3·75 mg/kg/day, 1·2 mg/kg/day and placebo). This allowed to minimize the potential effect of external factors over the course of the treatment and microdialysis. (ii) All the microdialysis experiments were performed at the same time points in the day, in order to avoid an effect of circadian status of the animal on neurotransmitter release. (iii) Over the period of aging and treatment, the cage location in the storage rack in animal facility was random and not known to the experimenter.

The experimenter was aware of individual animals allocation to the experimental groups over the whole period of the experiment, however was not personally participating in the work until microdialysis cannula implantation. The food containing different doses of anle138b was provided to the mice by veterinary technician who was not aware of the design, aim, and other details of the experiment. Cannula implantation and microdialysis was performed by the experimenter, but then, collected samples were submitted for HPLC measurements of dopamine to the collaborator, who was not aware of the allocation of the samples to the experimental groups. Next, the raw data were returned to the experimenter who analyzed them using defined algorithm.

Results:

This experiment showed that anle138b given in a daily dose of 11·1 mg/kg/day or higher is fully efficacious in rescuing the deficit in striatal dopamine release in this animal model. In total, up to 300 mg/kg/day were given and stabile maximal effects were observed (see figure). Based on the analysis of blood levels of anle138b after chronic dosing (14 days) using the same formulation (anle138b in food pellets), doses of ≥11·1 mg/kg/day correspond to an AUC_0-24_ exposure of ≥ 300 ng*h/ml.


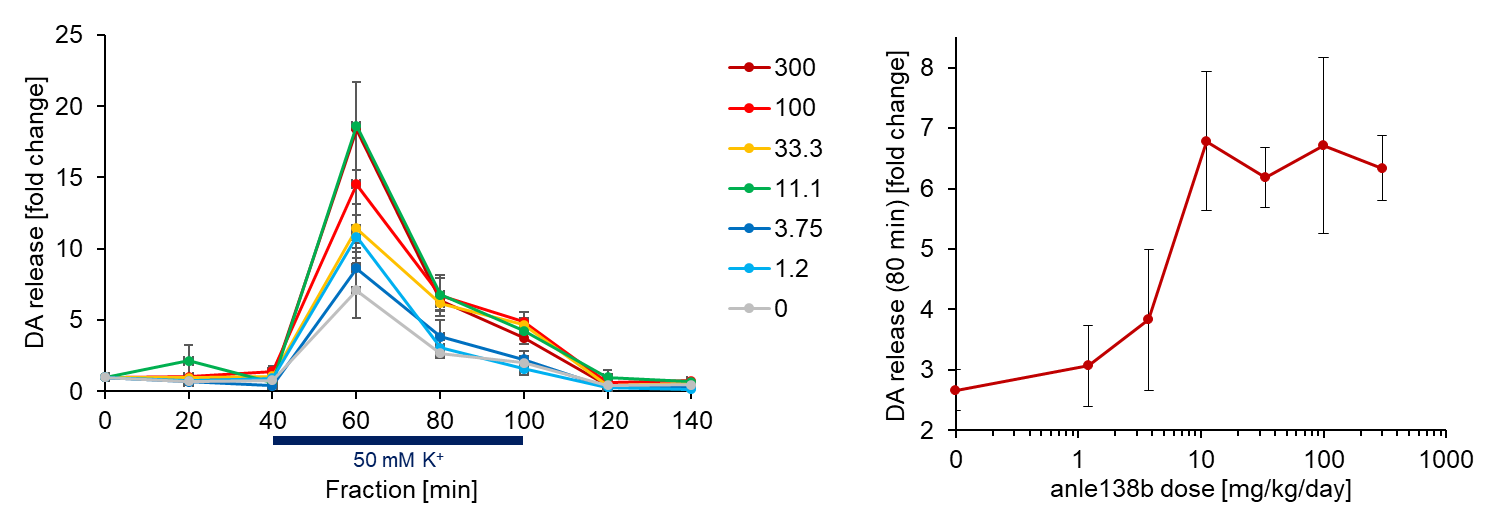


Figure Legend: (A) In a dose-response experiment, anle138b treatment was started at 6 months, i.e. when impairment of striatal DA release is already observed in MI2 mice (Wegrzynowicz et al., 2019). MI2 mice were fed with standard mouse chow supplemented with different doses of anle138b (0, 0·008, 0·025, 0·074, 0·222, 0·667, and 2 grams of compound per kg of food, respectively), provided at libitum. Assuming a daily food consumption of 0·15 g food pellets per g body weight, the doses were considered to correspond to 1·2, 3·75, 11·1, 33·3, 100, and 300 mg/kg/day. After 2 months of treatment, striatal release of DA was analysed following K^+^ stimulation using *in vivo* microdialysis, as previously reported (Wegrzynowicz et al., 2019). (B) Dose-response curve showing dopamine release at time point 80 minutes. 5-8 animals per dose were analyzed, shown are mean and standard error.

References:

Garcia-Reitböck P, Anichtchik O, Bellucci A, Iovino M, Ballini C, Fineberg E, Ghetti B, Della Corte L, Spano PF, Tofaris GK, Goedert M, Spillantini MG (2010) SNARE protein redistribution and synaptic failure in a transgenic mouse model of Parkinson’s disease. Brain 133:2032–2044

Paxinos G, Franklin KBJ (2004) The mouse brain in stereotaxic coordinates. Elsevier Academic Press, Amsterdam

Wegrzynowicz M, Bar-On D, Calo’ L, Anichtchik O, Iovino M, Xia J, Ryazanov S, Leonov A, Giese A, Dalley JW, Griesinger C, Ashery U, Spillantini MG (2019) Depopulation of dense α-synuclein aggregates is associated with rescue of dopamine neuron dysfunction and death in a new Parkinson’s disease model. Acta Neuropathologica 138: 575–595
