# Supplementary material for: Safety, tolerability and pharmacokinetics of the oligomer modulator anle138b with exposure levels sufficient for therapeutic efficacy in a murine Parkinson model: A randomised, double-blind, placebo-controlled phase 1a trial
Source: eBioMedicine. 2022 Apr 29;80:104021. doi: 10.1016/j.ebiom.2022.104021 (PMC9065877; doi:10.1016/j.ebiom.2022.104021)
Supplement: Supplementary file 2 [file mmc2.pptx]

## Slide 1
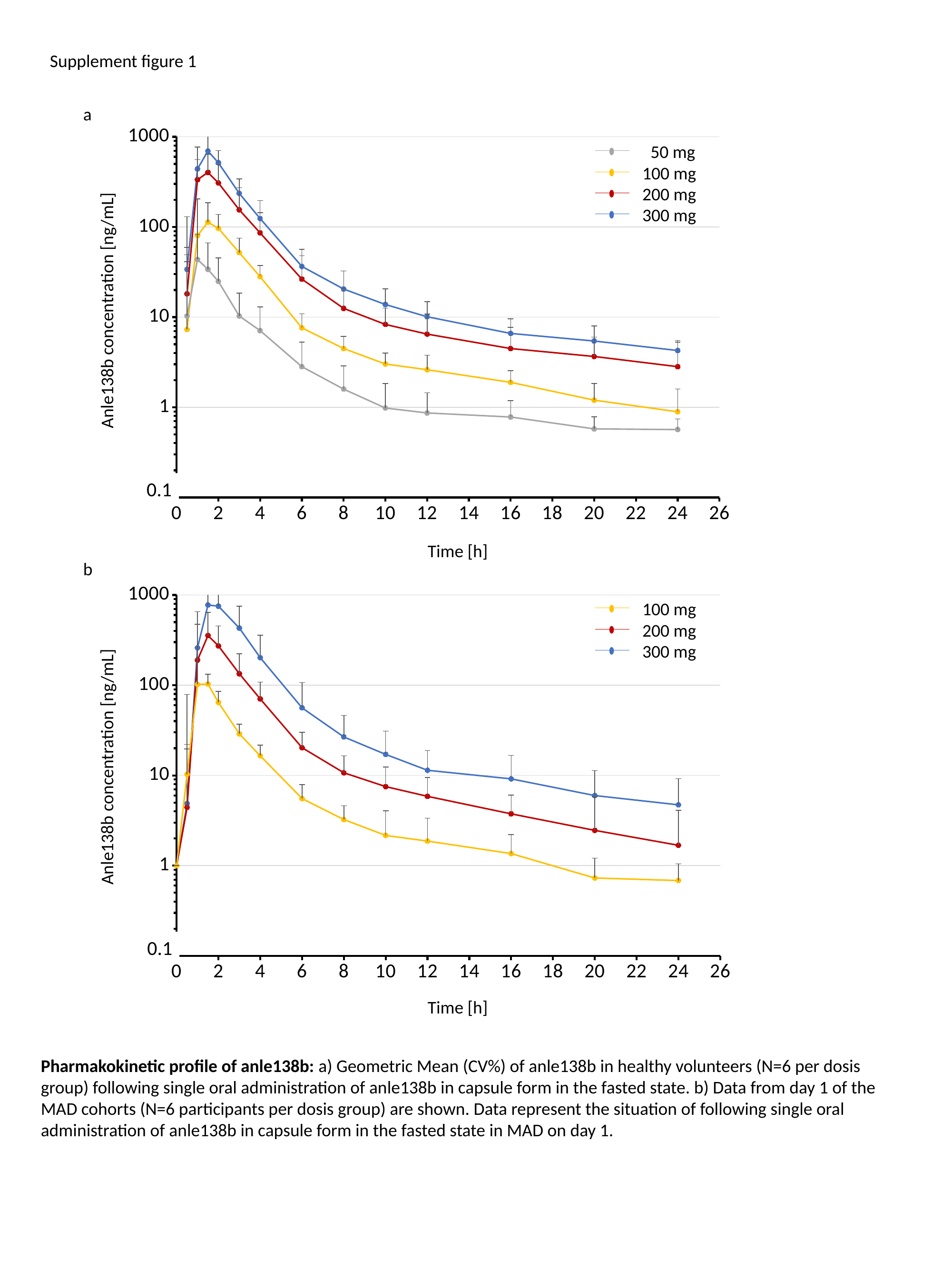

Supplement figure 1
a
### Chart
| Category | 50 mg | 100 mg | 200 mg | 300 mg |
|---|---|---|---|---| 50 mg
100 mg
200 mg
300 mg
Anle138b concentration [ng/mL]
0.1
Time [h]
b
### Chart
| Category | Mittelwert | Mittelwert | Mittelwert |
|---|---|---|---|100 mg
200 mg
300 mg
Anle138b concentration [ng/mL]
0.1
Time [h]
Pharmakokinetic profile of anle138b: a) Geometric Mean (CV%) of anle138b in healthy volunteers (N=6 per dosis group) following single oral administration of anle138b in capsule form in the fasted state. b) Data from day 1 of the MAD cohorts (N=6 participants per dosis group) are shown. Data represent the situation of following single oral administration of anle138b in capsule form in the fasted state in MAD on day 1.

## Slide 2
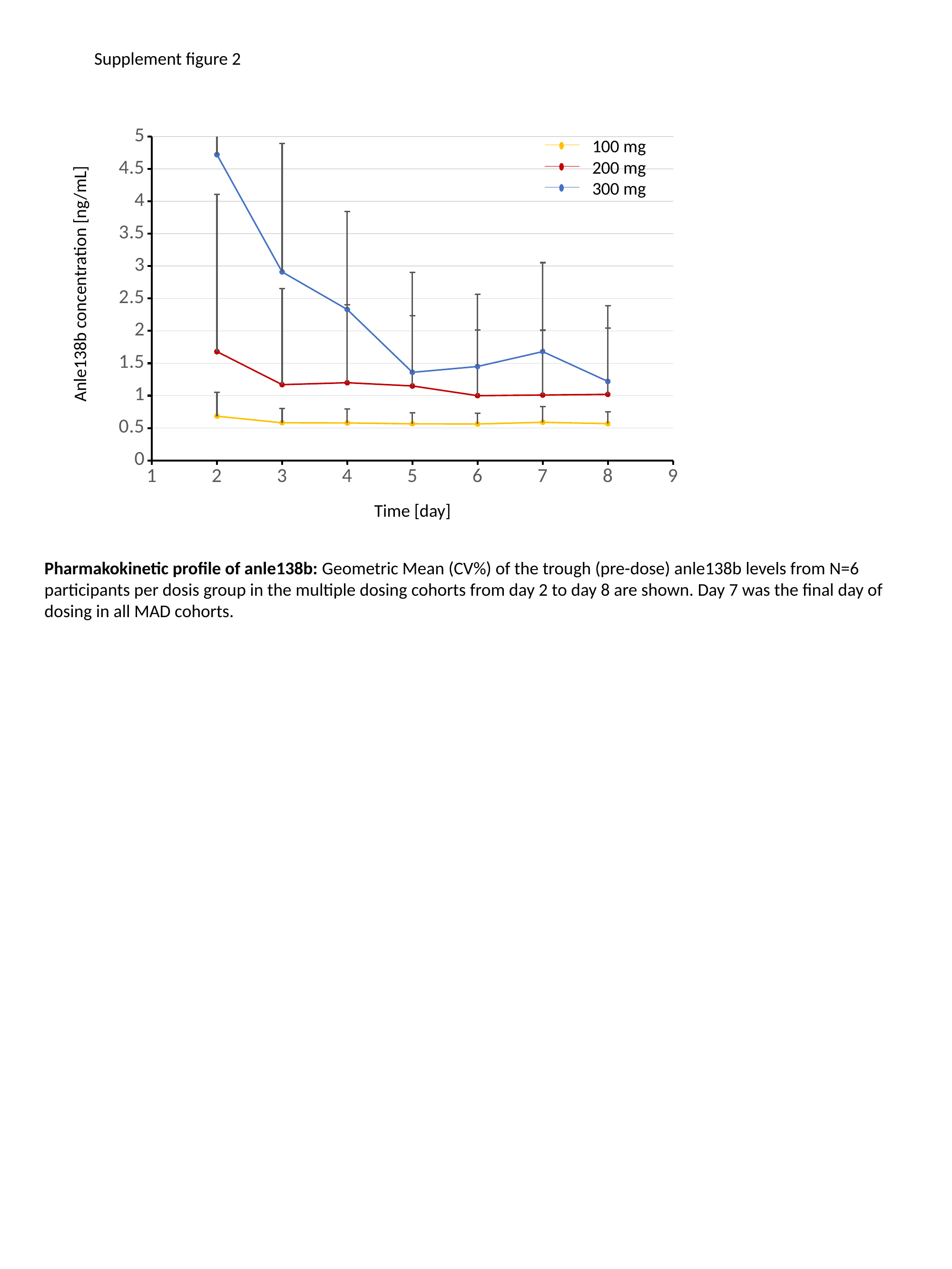

Supplement figure 2
### Chart
| Category | Mean 300mg | Mean 200mg | Mean 100mg |
|---|---|---|---|100 mg
200 mg
300 mg
Anle138b concentration [ng/mL]
Time [day]
Pharmakokinetic profile of anle138b: Geometric Mean (CV%) of the trough (pre-dose) anle138b levels from N=6 participants per dosis group in the multiple dosing cohorts from day 2 to day 8 are shown. Day 7 was the final day of dosing in all MAD cohorts.
